# The Rho-Family GTPase Rac1 Regulates Integrin Localization in Drosophila Immunosurveillance Cells

**DOI:** 10.1371/journal.pone.0019504

**Published:** 2011-05-16

**Authors:** Miguel J. Xavier, Michael J. Williams

**Affiliations:** Institute of Biological and Environmental Sciences, University of Aberdeen, Aberdeen, United Kingdom; Alexander Flemming Biomedical Sciences Research Center, Greece

## Abstract

**Background:**

When the parasitoid wasp *Leptopilina boulardi* lays an egg in a *Drosophila* larva, phagocytic cells called plasmatocytes and specialized cells known as lamellocytes encapsulate the egg. The *Drosophila* β-integrin Myospheroid (Mys) is necessary for lamellocytes to adhere to the cellular capsule surrounding *L. boulardi* eggs. Integrins are heterodimeric adhesion receptors consisting of α and β subunits, and similar to other plasma membrane receptors undergo ligand-dependent endocytosis. In mammalian cells it is known that integrin binding to the extracellular matrix induces the activation of Rac GTPases, and we have previously shown that Rac1 and Rac2 are necessary for a proper encapsulation response in *Drosophila* larvae. We wanted to test the possibility that Myospheroid and Rac GTPases interact during the *Drosophila* anti-parasitoid immune response.

**Results:**

In the current study we demonstrate that Rac1 is required for the proper localization of Myospheroid to the cell periphery of haemocytes after parasitization. Interestingly, the mislocalization of Myospheroid in Rac1 mutants is rescued by hyperthermia, involving the heat shock protein Hsp83. From these results we conclude that Rac1 and Hsp83 are required for the proper localization of Mys after parasitization.

**Significance:**

We show for the first time that the small GTPase Rac1 is required for Mysopheroid localization. Interestingly, the necessity of Rac1 in Mys localization was negated by hyperthermia. This presents a problem, in *Drosophila* we quite often raise larvae at 29°C when using the GAL4/UAS misexpression system. If hyperthermia rescues receptor endosomal recycling defects, raising larvae in hyperthermic conditions may mask potentially interesting phenotypes.

## Introduction

Research in the last fifteen years has led to significant breakthroughs providing evidence of a high degree of similarity between insect and mammalian innate immune responses and highlighted *Drosophila* as a model system for studying the evolution of innate immunity, both humoral and cellular [1]–[4] **.** When the morphology of *Drosophila* circulating immunosurveillance cells (haemocytes) is compared, three types of cells can be identified: plasmatocytes, crystal cells and lamellocytes. Plasmatocytes, similar to the mammalian monocyte/macrophage lineage, are professional phagocytes, dedicated to the phagocytosis of invading pathogens and apoptotic bodies. In healthy larvae they make up about ninety-five percent of circulating haemocytes and are involved in phagocytosis, encapsulation and the production of antimicrobial peptides. The other approximately five percent of circulating haemocytes in healthy larvae consists of crystal cells which rupture to secrete components of the phenol oxidase cascade, involved in melanization of invading organisms, wound repair and coagulation [2], [3], [5], [6]. The third cell type, known as lamellocytes, are rarely seen in healthy larvae and seem to be specialized for the encapsulation of invading pathogens [7]–[9].

Insects have an open circulatory system in which circulatory fluid is in a cavity known as the hemocoel. Endoparasitic wasps from the Hymenoptera family are known to parasitize *Drosophila* larvae by laying an egg within the hemocoel. Once a wasp egg is recognized as foreign circulating plasmatocytes somehow adhere to the invader. After spreading around the wasp egg plasmatocytes form cellular junctions between the cells [10], [11], effectively separating the egg from the hemocoel. Next, lamellocytes recognize the plasmatocytes surrounding the egg. The final phase of encapsulation involves melanization of the capsule due to crystal cell degranulation. From these events it is obvious that adhesion and cell shape change are an essential part of the *Drosophila*'s cellular immune response against parasitoid wasp eggs.

Previously, Irving et al. [12] published that a *Drosophila* β-integrin, Myospheroid, is necessary for lamellocytes to adhere to the cellular capsule surrounding the Leptopilina boulardi egg. Integrins are heterodimeric adhesion receptors consisting of α and β subunits. Like other plasma membrane receptors, integrins are known to undergo ligand-dependent endocytosis [13]–[17]. After endocytosis receptors can be recycled via two distinct mechanisms: a short-loop recycling pathway and a long-loop recycling pathway. Following internalization, receptors are delivered to early endosomes where decisions are made concerning the fate of the internalized receptors. Those selected for the short-loop pathway are sorted to early endosome subdomains and then under the control of Rab4 GTPase they are rapidly recycled to the plasma membrane [16], [17]. Alternatively, receptors may pass from early endosomes to the perinuclear recycling compartment (PNRC) and return to the plasma membrane via a long-loop recycling pathway. From these reports it becomes obvious that Rab GTPases are centrally important for integrin recycling. Like all small GTPases, Rabs cycle between GDP and GTP. In the GDP bound state Rab GTPases are recognized by Rab-αGDI which translocates them between membranes [18]. In mammalian cells the heat shock protein Hsp90 was shown to regulate Rab-αGDI-dependent retrieval of both Rab1 and Rab3A from membranes [19], [20], suggesting a link between chaperone proteins and Rab GTPase signaling.

In mammalian cells it is known that integrin binding to the extracellular matrix induces the activation of Rac GTPases [21], and we have shown that both *Drosophila* Rac1 and Rac2 are necessary for a proper encapsulation response [11], [22]. Also, to help the wasp embryo develop *L. boulardi* females inject various proteins able to inhibit the *Drosophila* immune response. One of these proteins encodes a RhoGAP able to inhibit Rac1 in *Drosophila* lamellocytes, making them less adhesive [23]–[25]. This led to the idea that *Drosophila* Racs are possibly acting downstream of Myospheroid in the cellular response against parasitization.

The current study presents the first evidence that in haemocytes, Myospheroid undergoes re-localization after parasitization. Furthermore, it shows that Rac1 and the heat shock protein Hsp83 are involved in regulating the proper cellular localization of Myospheroid.

## Results

### Myospheroid undergoes activation dependent endocytosis

**Figure 1:**
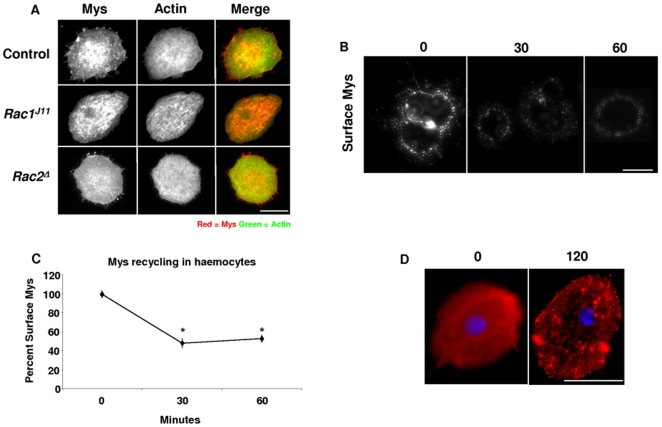
Myospheroid undergoes activation-dependent internalization in lamellocytes. (A) Lamellocytes were bled from at least 24 control (*w^1118^*), homozygous *Rac1^J11^*, and homozygous *Rac2^Δ^* larvae approximately 40 hours post-parasitization and stained for Myospheroid protein expression (red), as well as actin (green). (B) Anti-Mys antibody was bound to the surface of lamellocytes bled from control (*w^1118^*) larvae, followed by internalization for various times as indicated (n = 12 larvae/time-point). The cells were not permeabilized (C) Graph indicating percentage of surface Myospheroid expressed on control (*w^1118^*) haemocytes. Fluorescent intensity was measured using ImageJ to calculate the amount of cell surface Myospheroid on lamellocytes. Asterisk indicates significant difference (One-way Anova, *P*<0.01), errors bars indicate ± SE; n = 12 larvae/time-point, 0 minutes n = 1256 haemocytes, 30 minutes n = 1294, 60 minutes n = 1175). (D) Anti-Mys antibody was bound to the surface of lamellocytes bled from control (*w^1118^*) larvae, followed by internalization for 120 minutes as indicated (n = 12 larvae/time-point). Cells were permeabilized to allow for visualization of internalized Myospheroid. Size bars always indicate 20 µm.

To look for possible interactions between the *Drosophila* Rac GTPases (*Rac1* and *Rac2*) and *mys,* in the larval cellular immune response, haemocytes were bled from parasitized control (*w^1118^*) or Rac mutants and stained for Mys protein expression. Although the *Drosophila* Rac GTPases, *Rac1* and *Rac2*, are redundant during embryogenesis, we have already shown that this is not the case in larval haemocytes [11], [22]. In haemocytes from parasitized control larvae, or *Rac2^Δ^* null mutants, Mys was spread evenly across the cell surface, but in *Rac1^J11^* homozygous loss-of-function mutants Mys seemed to mislocalize (Figure 1A).

To begin to understand if Mys is internalized after activation, haemocytes were bled from non-parasitized third-instar larvae and incubated in normal *Drosophila* cell culture media that contained anti-Mys antibodies, for one hour at 4°C. The low temperature inhibits receptor internalization. After one hour cells were incubated at 25°C in fresh media, for the indicated time-points, to allow for Mys internalization (Figure 1B). Immunofluorescence microscopy of non-permeablized cells, revealed that Mys was evenly dispersed across the cell surface on some haemocytes, while on others, Mys had a more punctate distribution and was visible in extended filopodia (Figure 1B). The level of Mys on the outer surface of non-permeablized haemocytes was tracked by using ImageJ to measure fluorescent intensity, average Mys surface-expression at time zero was set at 100% (Figure 1C). After 30 and 60 minutes at 25°C, Mys cell surface expression was reduced in comparison to the 0 time-point. By 30 minutes, surface Mys levels were 49% (SE ± 7, p < 0.05) of initial expression, while at 60 minutes Mys surface expression was 53% (SE ± 5, p<0.05) of time 0 expression levels (Figure 1C). To show the reduction in Mys expression was due to relocalization, we examined the total cellular antibody-bound pool by staining permeabilized cells for Mys. Under these conditions, after 120 minutes Mys had accumulated in some compartment that was not observed at the 0 time-point (Figure 1D). These results indicate that Mys undergoes relocalization in haemocytes upon activation.

### Myospheriod and Rac1 interact during encapsulation

**Figure 2:**
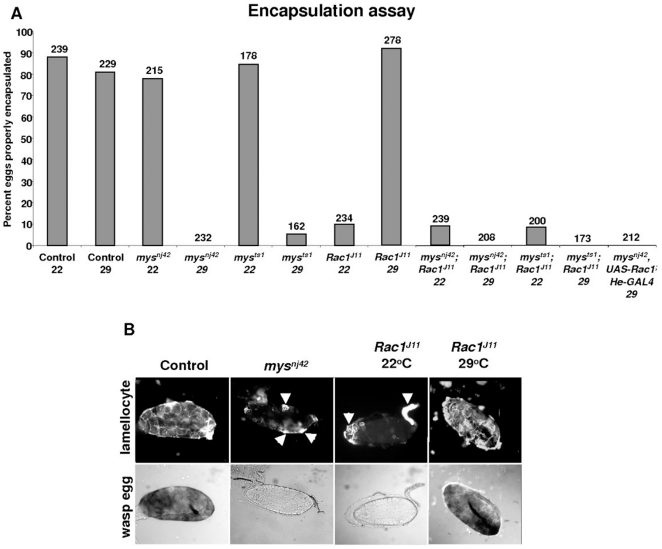
Myospheroid and Rac1 are necessary for encapsulation of *L. boulardi* eggs. (A) Encapsulation capacities of loss-of-function mutants in response to parasitization by *L. boulardi* G486. Values for proper encapsulation were calculated by the following equation [(Number of properly encapsulated wasp eggs/number of parasitized larvae) x 100]. Numbers above the bars indicate the number of wasp-parasitized larvae examined. (B) Wasp eggs collected 38–40 hours after parasitization were stained with the lamellocyte-specific antibody L1. Bright field pictures were taken to show that eggs recovered from the loss-of-function larvae were not properly melanized. Arrows indicate lamellocytes attached to wasp eggs recovered from either *mys^nj42^* or *Rac1^J11^* mutant larvae. 20-24 wasp eggs were dissected for staining from parasitized larvae of the various genotypes.

When the larval immune response reacts properly against eggs from the avirulent wasp strain *L. boulardi* G486, a darkened cellular capsule surrounding the egg is visible in the hemocoel 30–40 hours after parasitization. The temperature-sensitive alleles of *myospheroid* (*mys^nj4^* ^2^ and *mys^ts1^*) used in this study has a non-permissive temperature of 29°C, so we measured the encapsulation ability of larvae raised at 22°C and 29°C. Parasitized control (*w^1118^*) larvae reared at 22°C or 29°C had encapsulation rates of 88% or 81% respectively (Figure 2A). Similar to what was reported previously [12] at the permissive temperature 78% of *mys^nj42^* and 85% of *mys^ts1^* larvae properly encapsulated the wasp egg, while at the non-permissive temperature only 1% of *mys^nj42^* and 5% of *mys^ts1^* larvae encapsulated the egg, thus *myspheroid* is required for a proper encapsulation response.

In our previous study, we reported that *Rac1^J11^* mutants were inhibited in their ability to encapsulate eggs from the avirulent wasp strain *L. boulardi* G486 [22]. In that study all encapsulation experiments were performed at 24°C. Interestingly, raising homozygous *Rac1^J11^* larvae at 29°C prior to parasitization rescued the encapsulation defect (Figure 2A). While only 10% of homozygous *Rac1^J11^* larvae raised at 22°C properly encapsulated the wasp egg, larvae raised at 29°C had a 92% encapsulation rate (Figure 2A). To assess if hyperthermic rescue of the *Rac1* encapsulation defect requires *myospheroid,* we measured the encapsulation ability of *mys^nj42^; Rac1^J11^* and *mys^ts1^; Rac1^J11^* double-homozygous larvae at 22°C and 29°C. Similar to homozygous *Rac1^J11^* mutants raised at 22°C, *mys^nj42^; Rac1^J11^* larvae had an encapsulation rate of 9%, while 8% of *mys^ts1^; Rac1^J11^* larvae properly encapsulated the wasp egg. At 29°C wasp eggs were never properly encapsulated in either strain (Figure 2A). Finally, we overexpressed wild-type Rac1 (*UAS-Rac1*) in a *mys^nj42^* mutant background using the haemocyte specific driver *Hemese-GAL4* (*He-GAL4*) [26], and raised the larvae at 29°C to see if Rac1 hyperactivity was able to rescue loss of Mys. Overexpression of Rac1 was unable to rescue the *mys^nj42^* encapsulation defect (Figure 2A). From these results we conclude that hyperthermic conditions are able to compensate for the loss of Rac1 during the encapsulation response, and that Myospheroid is required for this hyperthermic rescue.

To gain further insight into the encapsulation defects of *mys^nj42^* and *Rac1^J11^* mutants, we compared the ability of control and homozygous mutant haemocytes to adhere to wasp eggs. Most wasp eggs dissected from *w^1118^* controls and homozygous *mys^nj42^* or *Rac1^J11^* mutants, raised at either 22°C or 29°C, were fully encapsulated by plasmatocytes (data not shown). By 38-40 hours post-parasitization, lamellocytes were observed completely surrounding wasp eggs recovered from control larvae. Yet, in *mys^nj4^* ^2^ mutant larvae reared at 29°C or *Rac1^J11^* mutants raised at 22°C, lamellocytes failed to completely surround the egg (Figure 2B, arrowheads). A similar result was observed for *mys^ts1^* larvae (data not shown). Rac1^J11^ mutants raised at 29°C where also completely surrounded by lamellocytes, though they had a rough appearance when compared to controls (Figure 2B), possibly due to a reduction in lamellocytes adhesion capabilities.

### Rac1 regulates Myospheroid cellular localization

**Figure 3:**
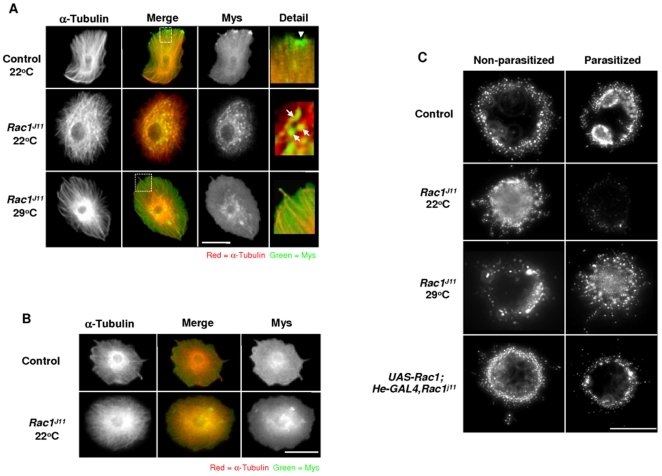
Myospheroid requires Rac1 for its proper localization. (A) Control (*w^1118^*) and homozygous *Rac1^J11^* larvae were raised at 22°C or 29°C, lamellocytes were recovered from at least 12 larvae during three experiments approximately 40 hours post-parasitization and stained for Myospheroid protein expression (green), as well as α-Tubulin (red). Arrowheads indicate Myospheroid protein localization. Size bar indicates 20 µm. (B) Lamellocytes were recovered from at least 24 non-parasitized late third instar larvae and stained for Myospheroid protein expression (green), as well as α-Tubulin (red). Size bar indicates 20 µm (C) Anti-Mys antibody was bound to the surface of lamellocytes bled from either non-parasitized or parasitized control (*w^1118^*), homozygous *Rac1^J11^*, or homozygous *UAS-Rac1;He-GAL4,Rac1^j11^* larvae raised at the indicated temperature, followed by internalization for 30 minutes (n = 12 larvae/time-point).

Myospheroid localization was compared in haemocytes bled from parasitized control or *Rac1^J11^* larvae raised at either 22 or 29°C. Although Myospheroid localization was perturbed in all haemocytes bled from parasitized *Rac1^J11^* larvae, we chose to focus our study on lamellocytes for the reason that their larger size and extremely spread morphology makes them more amenable for cell biology analysis. In lamellocytes recovered from control larvae, raised at either 22°C or 29°C, Myospheroid protein was observed accumulating at various places along the cell periphery (Figure 3A, see Detail, arrowhead). In parasitized *Rac1^J11^* larvae, raised at 22°C, instead of accumulating at the cell periphery, Myospheroid protein appeared to localize intracellularly, (Figure 3A, see Detail, arrows). Raising *Rac1^J11^* mutants at 29°C rescued their encapsulation defect, we examined lamellocytes to see if this could be due to Myospheroid localization. In lamellocytes recovered from parasitized *Rac1^J11^* larvae raised at 29°C, Mys localization was similar to controls.

Other studies have shown that integrin relocalization can be activation-dependent [17], [27]. To test if this was the case in the *Drosophila* cellular immune response, lamellocytes from non-parasitized control and homozygous *Rac1^J11^* larvae raised at 22°C were stained for Myospheroid protein expression. Unlike parasitized *Rac1^J11^*, in non-parasitized larvae Myospheroid was observed at the cell periphery (Figure 3B). To verify that Rac1 was regulating Mys localization, haemocytes were bled from third-instar larvae and an internalization assay was performed on non-permeabilized cells. In haemocytes bled from controls and non-parasitized *Rac1^J11^* larvae, Mys had a punctate plasma membrane distribution after 30 minutes at 22°C. In haemocytes from parasitized *Rac1^J11^* larvae, raised at 22°C, very little Mys was observed on the cell surface (Figure 3C), while in parasitized *Rac1^J11^* larvae raised at 29°C, Mys protein accumulated on the cell surface. In order to confirm that the Mys localisation defect was due to a lack of Rac1 signalling, we overexpressed *Rac1* in a homozygous *Rac1^J11^* background [26], [28]. In haemocytes recovered from these parasitized larvae, raised at 22°C, Mys localized to the cell surface (Figure 3C). From these results we conclude that Myospheroid localization in lamellocytes becomes Rac1-dependent only after immune activation.

### Rac1 localizes to microtubules in lamaellocytes

**Figure 4:**
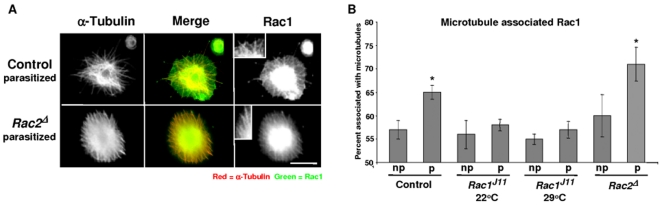
Rac1 localizes to microtubules in lamellocytes. (A) Lamellocytes were recovered from at least 24 control (*w^1118^*), homozygous *Rac1^J11^*, and *Rac2^Δ^* larvae approximately 40 h post-parasitization and stained for Rac (green) and α-Tubulin (red) protein expression. (B) ImageJ was used to measure fluorescent intensity of Rac1 localized microtubule staining from at least 50 haemocytes from three different larvae. An asterisk indicates a significant difference (error bars show ±SE, *Student's *t*-test, P<0.01) compared with the non-parasitized control strain.

To determine if Rac1 protein distribution in haemocytes correlated with the Mys mislocalization phenotype, lamellocytes were bled from larvae 38–40 hour post-parasitization, and stained for Rac1 and α-Tubulin expression. Fluorescent intensity of microtubule-associated Rac1, versus the rest of lamellocyte Rac1 expression, was calculated using ImageJ over/under threshold analysis. In lamellocytes, from parasitized or non-parasitized control larvae, much of the Rac1 staining co-localized with microtubules (Figure 4A and 4B). In lamellocytes, from non-parasitized controls, when microtubule-localized Rac expression was compared to total cellular Rac1 levels, ∼57% (SE ± 2) of the expression was concentrated to microtubules (Figure 4B). After parasitization, the amount of microtubule-associated Rac1 expression increased to ∼65% (SE ± 1.5). When Rac1 protein distribution was analyzed in *Rac1^J11^* mutants, there seemed to be little increase in microtubule-associated Rac1 after parasitization (Figure 4B). The remnant Rac1 microtubule staining in *Rac1^J11^* mutants could be due to the expression of Rac2 in lamellocytes. To see if this was true, the distribution of Rac1 in non-parasitized and parasitized *Rac2^Δ^* null mutants was compared. These mutants do not express Rac2 protein [29], [30], so all Rac staining observed should be due to the expression of Rac1. Similar to what was observed in control larvae, after parasitization there was an increase in the amount of microtubule-associated Rac1, ∼60% (SE ± 4.5) in non-parasitized and ∼71% (SE ± 4) in haemocytes from parasitized larvae (Figure 4A and 4B). From these results we conclude that Rac1 co-localizes with microtubules in lamellocytes, and the amount of microtubule-associated Rac1 increases after parasitization.

### Hsp83 overexpression rescues Myospheroid mislocalization

**Figure 5:**
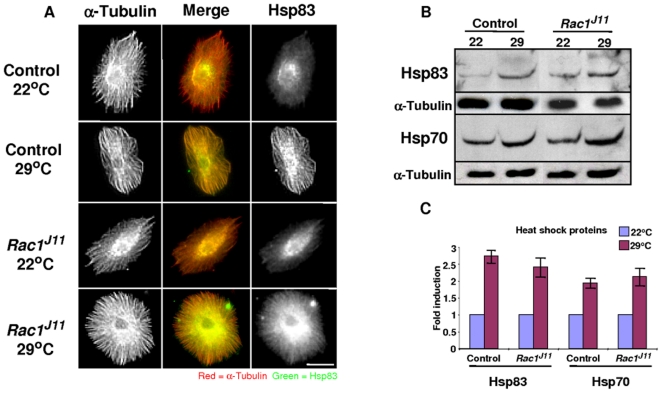
Hsp83 localizes to microtubules in lamellocytes. (A) Control (*w^1118^*) and homozygous *Rac1^J11^* larvae were raised at 22°C or 29°C, lamellocytes were recovered from at least 24 larvae approximately 40 h post-parasitization and stained for Hsp83 protein expression (green), as well as α-Tubulin (red). (B) Western analysis to look at Hsp83 and Hsp70 protein levels was performed on protein collected from control (*w^1118^*) and homozygous *Rac1^J11^* larvae were raised at 22°C or 29°C, α-Tubulin was used as a loading control. (C) Quantification of the Western analysis comparing expression levels of Hsp83 or Hsp70 in lysates from whole third instar larvae raised at either 22°C or 29°C (n = 5, error bars show ±SE, * indicates Student's *t*-test, P < 0.01).

Rac1-dependent localization of Mys was overcome by hyperthermia, leading us to speculate that *Drosophila* heat shock proteins might be involved. To begin to investigate the possibility that a heat shock protein was involved in hyperthermic rescue of Mys mislocalization, lamellocytes from parasitized larvae were stained for the *Drosophila* homologue of Hsp90, known as Hsp83. In lamellocytes bled from parasitized control and *Rac1^J11^* larvae, raised at 22°C, Hsp83 localized to a perinuclear region and a low-level of expression was also observed along the microtubules. Much more Hsp83 protein co-localized with microtubules when control and *Rac1^J11^* mutants were raised at 29°C prior to parasitization (Figure 5A). This increase of Hsp83 could be due to a redistribution of protein from the perinuclear region to microtubules, or it could be due to an overall increase in Hsp83 expression. Protein from whole wandering third-instar larvae was collected and Western analysis was performed to see whether raising the larvae at 29°C was sufficient to increase total Hsp83. As shown in Figure 5B, raising control or *Rac1^J11^* larvae under hyperthermic conditions was sufficient to increase Hsp83 expression levels. In controls or *Rac1^J11^* mutants there was about a 2.5 fold increase in total Hsp83 protein respectively when larvae were raised at 29°C. Comparatively there was a 2 fold increase in total Hsp70 protein when larvae were raised a 29°C (Figure 5C).

**Figure 6:**
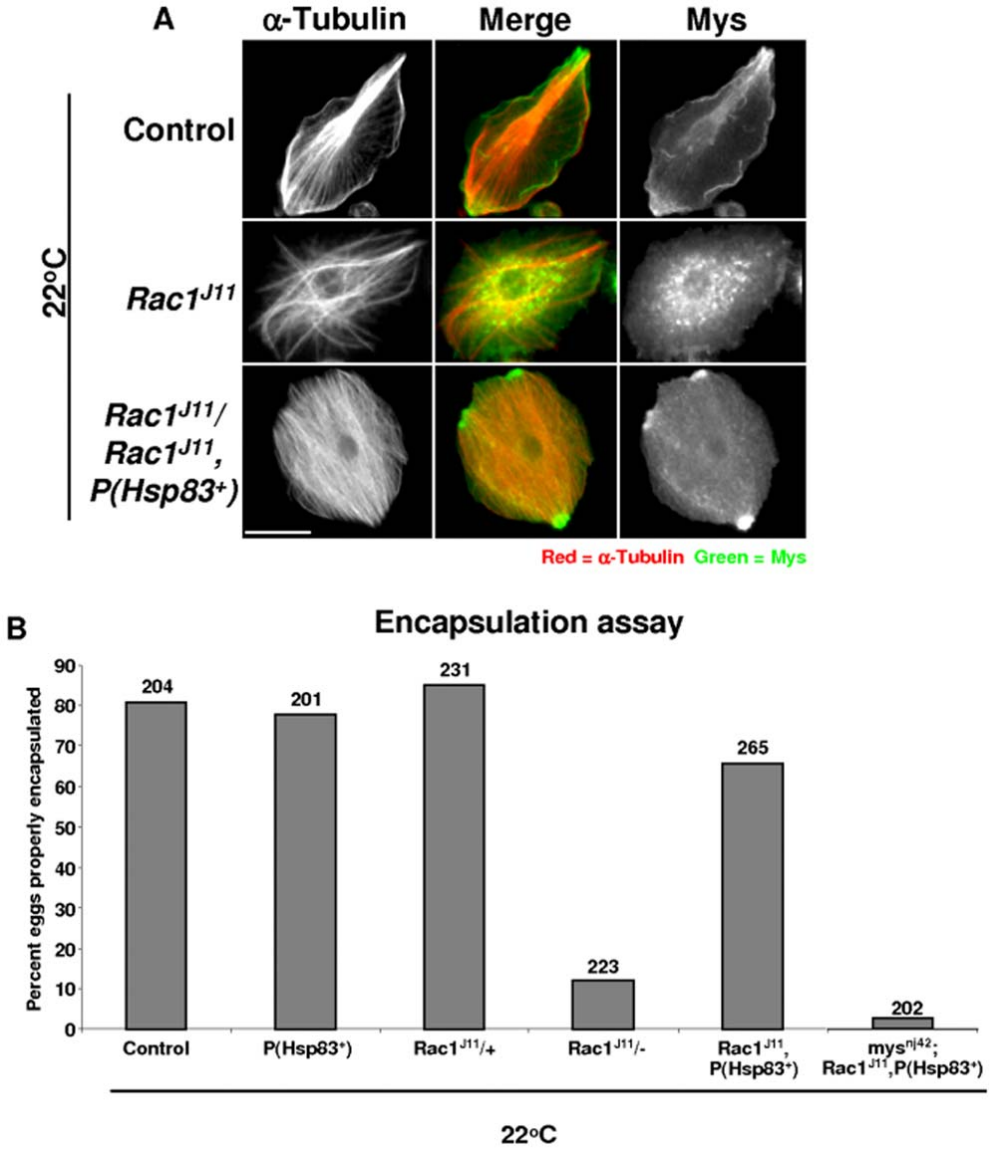
Hsp83 overexpression rescues loss of Rac1 signal. (A) Control (*w^1118^*), homozygous *Rac1^J11^,* homozygous *Rac1^J11^,P(Hsp83+)* larvae were raised at 22°C, lamellocytes were recovered from at least 24 larvae approximately 40 hours post-parasitization and stained for Myospheroid protein expression (green), as well as α-Tubulin (red). Size bar indicates 20 µm. (B) Control (*w^1118^*), homozygous *Rac1^J11^,* homozygous *Rac1^J11^,P(Hsp83+)*, and *mys^nj42^;Rac1^j11^,P(Hsp83)* larvae were raised at 22°C and an encapsulation assay in response to parasitization by *L. boulardi* G486 was performed. Values for proper encapsulation were calculated by the following equation [(Number of properly encapsulated wasp eggs/number of parasitized larvae) x 100]. Numbers above the bars indicate the number of wasp-parasitized larvae examined.

Finally, Hsp83 was overexpressed in *Rac1^J11^* mutant larvae to test the idea that increased Hsp83 activity could rescue the Mys localization defect. Adding a wild-type Hsp83 transgene was sufficient to rescue the Mys localization defect in parasitized *Rac1^J11^* homozygous mutant larvae raised at 22°C (Figure 6A). Hsp83 overexpression was also able to increase the encapsulation rate to 68% at 22°C in a *Rac1^J11^* mutant background. Finally, overexpression of Hsp83 failed to rescue the encapsulation defect in a *mys^nj42^;Rac1^J11^* mutant background (Figure 6B).

## Discussion

Although it is known that integrins and Rho-family GTPases are involved in cellular adhesion, little is known about how these proteins interact in circulating immunosurveillance cells [31]–[34]. In *Drosophila* the β-integrin *myospheroid* is necessary for lamellocytes to properly encapsulate parasitoid wasp eggs [12]. Also, we have previously published the involvement of the Rho-family GTPases Rac1 and Rac2, in a non-redundant fashion, in the cellular immune response against the parasitoid wasp *Leptopilina boulardi* [11], [22]. Here, we present evidence that the Rho-family GTPase *Rac1* and the heat-shock protein Hsp83 are necessary for the proper localization of the β-integrin Myospheroid in lamellocytes during the *Drosophila* encapsulation response.

Requisite integrin recycling during cellular adhesion is becoming increasingly recognized [17], [32]. Like many other transmembrane receptors, when integrins become activated they go through a process of endocytosis. After endocytosis integrins travel to early-endosomes where they are sorted, and are either directly recycled back to the plasma membrane via Rab4-regulated vesicles or to a compartment known as the perinuclear-recycling center (PNRC) [13]–[17], [35]. In wild-type haemocytes prior to parasitization the *Drosophila* β-integrin Myospheroid is observed evenly distributed at the plasma membrane. After cellular activation by parasitization, or by crosslinking due to the addition of Mys-specific antibodies, Myospheroid re-localizes to various places around the cell periphery, including filopodial extensions. This localization fits with the idea of why integrin recycling is important. Basically, integrins go through endocytosis at the retracting end of a cell and are recycled to the leading edge where they undergo exocytosis [15], [17]. This recycling provides fresh integrin receptors to the leading edge of the cell. During the encapsulation of parasitoid wasp eggs, integrin recycling may be necessary for proper cellular spreading and adhesion, again integrin would be needed at the periphery of the spreading cells.

In *mys* and *Rac1* mutants, lamellocytes fail to properly encapsulate the parasitoid wasp egg. Interestingly, in *mys* mutants, plasmatocytes adhere and spread around the egg similar to control larvae, showing that *mys* function is not required in plasmatocytes during the encapsulation process. Mys is expressed at much higher levels in lamellocytes than in plasmatocytes and recently we have shown that another *Drosophila* β-integrin, βυ, is required for plasmatocytes to adhere to *L. boulardi* eggs (data not shown, and M. Williams manuscript in preparation). This leads to the possibility of different integrin receptors being necessary to varying degrees in different haemocyte cell types. Unfortunately, since lamellocytes adhere to plasmatocytes and not to the wasp egg, we cannot judge the necessity of βυ for lamellocyte function during the encapsulation process.

Lamellocytes from *Rac1* mutants raised at 29°C are able to adhere to the cellular capsule surrounding the wasp egg in a Mys dependent manner, and hyperthermic conditions rescued Rac1-depedent Mys localization. Previously, it was demonstrated in mammalian cell lines that Hsp90 was involved in the proper localization of a RabGDI, necessary for Rab GTPase recycling [19], [20]. The basal Hsp83-regulated Rab GTPase dependent recycling apparatus may not be able to cope with the large turnover of activated Mysopheroid after parasitization, and thus a second Rac-regulated pathway is induced. Raising the larvae under hyperthermic conditions increases the amount of Hsp83 in the lamellocytes and may increase the efficiency of the basal Rab GTPase endosomal-recycling apparatus. This could account for the hyperthermic rescue of Mys localization in Rac1 mutant larvae.

The addition of PDGF to 3T3 fibroblast cells induces Rab4-dependent integrin recycling, and this recycling requires the serine-threonine kinase PKD1 [13], [14]. Furthermore, PKD1 activates JNK in a Rac1 dependent manner [36]. In *Drosophila* the PDGF/VEGF receptor PVR signals via Rac1 to activate JNK for thoracic closure during metamorphosis and for border cell migration during oogenesis [37], [38]. PVR is also known to be necessary for haemocytes to migrate during embryogenesis [39], [40], and Pvr signalling is sufficient to induce the larva immune response [26]. There are three Pvr ligands in *Drosophila*, Pvf1, -2, and -3, and in larvae it has been shown that overexpression of Pvf2 is also sufficient to activate the cellular immune response [41]. This leads to the possibility that similar to 3T3 cells Pvr functions via Rac1 and JNK to control Rab4-regulated integrin recycling in lamellocytes after parasitization. Though Pvr has been shown to be involved in larval haematopoiesis, it must be mentioned that it is not known whether Pvr, or any of its ligands, is actually involved in the anti-parasitoid cellular immune response.

Finally, when the wasp *L. boulardi* parasitizes *Drosophila* larvae it also injects venom, one of the proteins the female wasp injects encodes a Rac-specific RhoGAP, called LbGAP [23]. To turn off GTPase signalling GTP-bound small GTPases, such as Rac1, are recognized by GTPase activating proteins (GAP) that accelerate GTP to GDP hydrolysis. Somehow LbGAP is able to localize to the cytosol of lamellocytes and is sufficient to inhibit lamellocytes from adhering to the growing cellular capsule surrounding the wasp egg [23], [23]–[25]. Considering the data presented in the current study, it is possible that LbGAP inhibits Rac1 in lamellocytes, thus inhibiting Myospheroid recycling after parasitization, making them less adherent and lowering their ability to properly encapsulate the wasp egg.

## Materials and Methods

### Insects

*Drosophila* strains were obtained from the Bloomington Stock Center, and the references are given in Flybase. *UAS-Rac1^IR^* flies were provided by Ryu Ueda [19], [20]. The *Hemese-GAL4* driver line was described previously [26]. Flies were kept on a standard cornmeal diet at 25°C. The G486 strain of *L. boulardi* was bred on a *w^1118^* stock of *Drosophila melanogaster* at room temperature. Adult wasps were maintained at room temperature on grape juice agar.

### Myospheroid internalization assay

Mys localization in haemocytes was followed by staining cells according to following protocol (modified from [35]). Haemocytes were bled from larvae into room temperature Schneider's media (Invitrogen) and allowed to adhere to a glass slide (SM-011, Hendley-Essex, Essex, UK) for 1 hour at 25°C. Haemocytes were stained with 4°C anti-integrin betaPS antibody (Developmental Studies Hybridoma Bank) diluted to 10 µg/ml in Schneider's media and incubated at 4°C for 1 hour. Afterwards cells were washed twice with cold Schneider's media. Immediately, the 0 time-point cells were fixed for 5 minutes with 3.7% paraformaldehyde/Phosphate Buffered Saline (PBS) at 4°C. Pre-warmed (25°C) Schneider's media was added to the other cells to stimulate recycling; these cells were fixed after 30 and 60 minutes. Cells were washed three times for 5 minutes with PBS, and then blocked for 30 minutes at room temperature with 3% BSA/PBS (Blocking solution). Cells were then incubated at room temperature for 1 hour with secondary antibody diluted in blocking solution, anti-mouse Alexo-fluor 488 (1∶500) (Invitrogen). Cells were washed three times for 5 minutes with PBS, followed by fixation with 3.7% paraformaldehyde/1x PBS for 5 minutes, and washed again three times with PBS for 5 minutes. Cells were mounted in 50% glycerol/1x PBS. Movement of integrin was further investigated by changing a step on the previous technique allowing for the visualisation of integrin inside the cells, for this purpose, after the initial fixation, cells were washed for 5 minutes each with PBS, PBX (0.1% Triton X-100 in 1x PBS) and a final wash with PBS. Cells were visualized using a Zeiss Axiovert 200 M epifluorescent microscope and digital pictures were taken with a Hamamatsu C4742-80-12AG video unit, controlled by the Simple PCI 6.1 program (Hamamatsu). ImageJ (NIH) was used for digital editing and for measuring fluorescent intensity.

### Wasp egg encapsulation assay

The encapsulation assay was done according to Sorrentino et al. [42]. Briefly, two days before parasitization the appropriate fly strains were crossed and kept at 21–25°C. Four or five females of *L. boulardi* G486 were allowed to infest at room temp for 2 h, after which the *Drosophila* larvae were transferred to apple juice plates and left at room temperature for 40–42 h. After this time the larvae were collected, washed in PBS, and then viewed under a stereomicroscope for the presence of a dark capsule. Larvae in which no dark capsule was observed were dissected in 20 µl of PBS to determine if they had been parasitized. Larvae containing eggs from the parasitoid that hadn't darkened by this time were scored as non-encapsulated. Non-parasitized larvae were excluded from the count.

### Antibodies and reagents

Mouse monoclonal anti-Myospheroid (Developmental Studies Hybridoma Bank, Iowa, USA), lamellocyte-specific mouse monoclonal antibody (L1a) [28] and plasmatocyte-specific monoclonal mouse anti-Nimrod [28], [43] were all used undiluted, mouse monoclonal anti-Rac1 (BD Biosciences) diluted 1∶250, mouse monoclonal antibody anti-α-Tubulin (Sigma) diluted 1∶1,000, rabbit polyclonal anti-α-Tubulin (Abcam) diluted 1∶500, rat monoclonal anti-Hsp90 (Abcam) diluted 1∶300.

### Immunofluorescence

#### Wasp egg staining

Wasp eggs were bled from larvae into PBS and allowed to attach to a glass slide for 5 minutes at room temperature. Staining and analysis were done according Williams et al. [11].

#### Circulating haemocyte staining

For all haemocyte antibody-staining, haemocytes were bled from a larva into PBS, and allowed to attach to a glass slide (SM-011, Hendley-Essex, Essex, UK) for 1 hour. Staining and analysis were done according to Williams et al. [22]. Cells were visualized using as in Myospheroid internalization assay section.

#### Measuring Rac1 concentration

The over/under threshold capability of ImageJ was used to measure fluorescence intensity of Rac1 staining localising to microtubules. The percent of Rac1 localising to microtubules was defined as the intensity of Rac1 staining along microtubules divided by total Rac1 staining of an individual haemocyte. At least 50 haemocytes from three different larvae were measured. For statistics, an initial ANOVA analysis (http://www.physics.csbsju.edu/stats/anova.html) indicated that parasitization affected rac1 localisation significantly. Multiple Student's *t*-tests (Microsoft Excel and http://www.graphpad.com/quickcalcs/ttest1.cfm) were performed to study specific interactions between genotypes, and in the case of UAS-RNAi their corresponding crosses, before and after parasitization.

### Western analysis

Wandering third instar larvae were collected and homogenized in cell lysis buffer (50 mM Tris/HCl pH 7.5, 10 mM MgCl_2_, 0.3 M NaCl, 2% IGEPAL). The lysate was microcentrifuged at 8000 xg for 5 minutes at 4°C and the supernatant recovered to a new tube. The concentration was then quantified using the BioRad DC Protein Assay Kit (BioRad). Protein fractions (10 mg) were diluted with Laemeli buffer and then heated at 65°C for 10 min. Samples were electrophoresed on a 4% polyacrylamide stacking gel, 12% resolving gel with appropriate size markers. Gels were washed three times for 10 min with transfer buffer (25 mM Tris, 190 mM glycine, 20% methanol), then transferred to a Hybond nitrocellulase membrane (Amersham). Membranes were blocked for 1 h at room temperature in Western blocking solution (1x Tris-buffered saline, 0.1% Tween-20 (TBST) with 5% non-fat dry milk). The rat anti-Hsp83 (Abcam) was diluted 1∶1000, the mouse anti-Hsp70 (Abcam) was diluted 1∶5,000, and the rabbit anti-α-Tubulin (Abcam) was diluted 1∶1000 in Western blocking solution and incubated with the membranes overnight at 4°C. Membranes were washed three times 10 minutes with 1x TBST. HRP-conjugated anti-rat secondary antibody (Abcam) was diluted 1∶5000, anti-mouse and anti-rabbit secondary (Amersham) were diluted 1∶10000 in Western blocking solution and incubated with the membranes at room temperature for 1 hour. Membranes were washed six times 10 minutes with 1x TBST and two times 5 minutes with 1x TBS. Finally, blots were incubated with ECL Plus Western blotting detection reagent (Amersham) for 5 minutes. Blots were exposed to X-ray film (Hyperfilm ECL, Amersham) for 60-120 seconds. Western analysis was repeated four times. The relative amount of antibody binding was evaluated by ImageJ.

### Statistical Analysis

The influence of time or of Rac1 on integrin recycling by haemocytes was analysed by ANOVA's General Linear Model allowing investigating both the effects of the genotype and time on the levels on the outer-surface of haemocytes, as well as the interaction of these factors. Tukey Simultaneous Tests were used to obtain pairwise comparisons of the different parameters means. All ANOVA and Student *t*-test analysis were conducted using the statistical analysis package provided in Minitab15.
